# Upregulated expression of DIXDC1 in intestinal-type gastric carcinoma: co-localization with β-catenin and correlation with poor prognosis

**DOI:** 10.1186/s12935-015-0273-2

**Published:** 2015-12-18

**Authors:** Lei Wang, Cong Tan, Fan Qiao, Weige Wang, Xiangnan Jiang, Peng Lian, Bin Chang, Weiqi Sheng

**Affiliations:** Department of Pathology, Fudan University Shanghai Cancer Center, Shanghai, 200032 China; Department of Oncology, Shanghai Medical College, Fudan University, Shanghai, 200032 China; Department of Cardiothoracic Surgery, Changhai Hospital, Second Military Medical University, Shanghai, 200433 China; Department of Colorectal Surgery, Fudan University Shanghai Cancer Center, Shanghai, 200032 China

**Keywords:** Gastric carcinoma, Intestinal-type, DIXDC1, β-catenin, Immunohistochemistry

## Abstract

**Background:**

DIXDC1 (Dishevelled-Axin domain containing 1) is a positive regulator of the Wnt pathway. In the field of cancer research, the role of DIXDC1 is unclear. Our previous in vitro study showed that DIXDC1 enhances β-catenin nuclear accumulation in gastric cancer cell lines. The aim of this study was to detect the expression of DIXDC1 in different histological subtypes of gastric carcinoma and to evaluate the correlation between the expression of DIXDC1 and β-catenin localization and clinicopathological parameters, including patients’ survival.

**Methods:**

Immunohistochemical staining was performed to characterize the expression of DIXDC1 and β-catenin in archived materials from 259 cases of gastric carcinoma. The χ2 test and the Fisher’s test were used to analyze correlations between DIXDC1 expression, β-catenin localization, and clinicopathological parameters. Univariate analyses were performed using the Kaplan–Meier method, and the survival difference between groups was assessed by the log-rank test. Multivariate analysis was performed using the Cox proportional hazards regression model.

**Results:**

Positive DIXDC1 staining was detected in tumor cells in 123 of 259 (47.5 %) cases. DIXDC1 expression in gastric carcinoma was significantly correlated with the histological intestinal-type (*P* < 0.001), the depth of tumor invasion (*P* < 0.001) and the lymph node metastasis (*P* = 0.006). In the intestinal-type, DIXDC1 was correlated with the nuclear and cytoplasmic β-catenin expression (*P* = 0.002). Kaplan–Meier analysis indicated that patients with high DIXDC1 expression had poor disease-specific survival (*P* < 0.001), especially in the intestinal-type. Moreover, multivariate regression analysis showed that positive expression of DIXDC1 was an independent prognostic predictor of intestinal-type gastric carcinoma.

**Conclusion:**

Our study indicated that DIXDC1 is a significant independent prognostic indicator in intestinal-type gastric carcinoma that plays an important role in carcinogenesis and progression of gastric carcinoma through the Wnt signaling pathway.

## Background

Gastric carcinoma (GC) is one of the leading causes of cancer-related death, especially in Asia. Gastric carcinogenesis can be considered a multistep process that involves specific genetic alterations. Dysregulation of Wnt signal transduction plays an important role in gastric carcinogenesis, especially in the development of intestinal-type gastric carcinoma [1–6].

DIXDC1 is the human homolog of Ccd1 (Coiled-coil-Dishevelled-Axin1), a DIX (Dishevelled-Axin) domain-containing protein and a positive regulator of the Wnt pathway in zebrafish neural patterning [7]. In the Wnt signaling pathway, the DIX domain is involved in both homomeric and heteromeric complexes between Axin and Dishevelled (Dvl) that form the multiprotein complexes of adenomatous polyposis coli (APC), glycogen synthase kinase 3 beta (GSK-3β), and β-catenin, which regulate T-cell-factor (TCF) signaling [8, 9]. In the field of cancer research, the role of DIXDC1 is unclear. Recent studies have shown that overexpression of DIXDC1 can increase the proliferation of colon cancer cells [10] and the invasion and migration ability of non-small cell lung cancer (NSCLC) [11], both of which are affected by the PI3 K/AKT pathway. Our previous study showed that DIXDC1 is overexpressed in gastric cancer (37/66) and that the upregulation of DIXDC1 is associated with poor prognosis. We also found that DIXDC1 promotes gastric cancer cell invasion and metastasis through the activation of the Wnt pathway [12].

Beta-catenin is a key factor of the Wnt pathway that is phosphorylated by GSK-3β and then degraded by the ubiquitin–proteasome system. Accumulation of cytoplasmic β-catenin results in its entry into the nucleus where it binds to the members of the TCF family and activates downstream target genes, such as c-myc and cyclin D1, which are involved in cell proliferation [13–17]. However, the regulatory mechanism of β-catenin stabilization is not yet completely understood. Our previous study showed that DIXDC1 enhances β-catenin nuclear localization by decreasing the phosphorylation level of β-catenin [12]. All of the results of in vitro experiments are needed to further certify in gastric carcinoma tissues. Therefore, the goals of this study are (a) to enlarge the sample size (259 cases) to further study the relationship between DIXDC1 expression and clinicopathological features of gastric carcinoma and its effect on patients’ prognoses; (b) to explore the expression pattern of DIXDC1 and the subcellular localization of β-catenin in different histopathological subtypes of gastric carcinoma (intestinal-type and diffuse-type); and (c) to verify the contribution of DIXDC1-mediated Wnt activation in intestinal-type gastric carcinogenesis.

## Results

### Patient characteristics

The mean and median age of these patients was 59.2 and 59.0 years old (range 30–87 years), respectively, and the male/female ratio was 3:1. Tumor size ranged from 0.4 to 14 cm (mean, 3.9 cm; median, 3.5 cm). According to the tumor-node-metastasis (TNM) staging system of the American Joint Committee on Cancer (AJCC, 7th edition), 163 cases (62.9 %) were in disease stage I/II and 96 cases (37.1 %) were in disease stage III/IV. Lymph node metastasis was observed in 154 cases (59.5 %). This cohort of gastric carcinomas was comprised of 151 cases of intestinal-type, 79 cases of diffuse-type, 25 cases of mixed-type and 4 cases of indeterminate-type. Follow-up information was available on 195 patients and ranged from 2 to 96 months (median, 47 months). At the end of the follow-up period, 87 patients were still alive and 108 had died of the disease. Disease-specific survival (DSS) at 1, 3, and 5 years was 98.8, 90.3 and 27.8 %, respectively.

### DIXDC1 expression in gastric carcinoma and its correlation with clinicopathological features

**Fig. 1:**
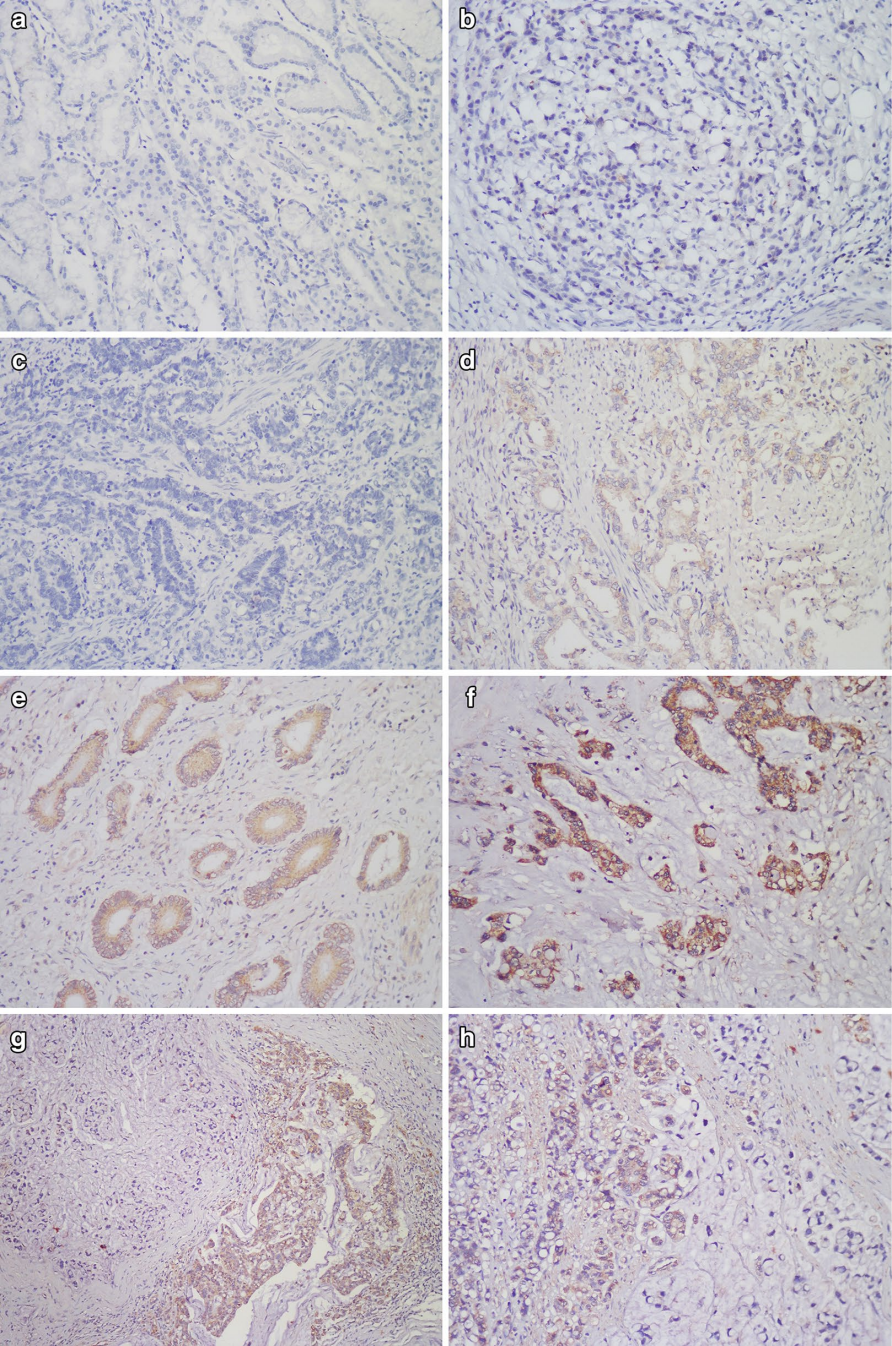
Immunohistochemical staining of DIXDC1 in gastric carcinoma. **a** Normal gastric mucosa with negative DIXDC1 expression. (×200) **b** Diffuse-type GC with negative DIXDC1 expression. (×200) **c** Intestinal-type GC showing negative DIXDC1 expression. (×200) **d** Intestinal-type GC showing mild cytoplasmic staining of DIXDC1 (scored as 1). (×200) **e** Intestinal-type GC with moderate cytoplasmic DIXDC1 staining (scored as 2). (×200) **f** Intestinal-type GC with intense cytoplasmic DIXDC1 staining (scored as 3). (×200) **g** (×100) **h** In mixed-type cases, positive DIXDC1 expression was observed only in the glandular formation areas, but not in the diffuse regions. (×200)

DIXDC1 was detected mainly in the cytoplasm of the tumor cells and displayed a diffuse pattern. Positive DIXDC1 staining was detected in 123 cases (47.5 %) of gastric carcinoma. Normal gastric mucosa was negative for DIXDC1 staining (Fig. 1). The relationship between DIXDC1 expression and histopathological characteristics is summarized in Table 1. Positive DIXDC1 staining was detected in 97 of 151 cases of intestinal-type (64.2 %), 13 of 79 cases of diffuse-type (16.5 %), 12 of 25 cases of mixed-type (48.0 %), and 1 of 4 cases of indeterminate-type (25.0 %) of gastric carcinoma. In some cases of mixed-type, positive DIXDC1 expression was only observed in the glandular areas, but not in the diffuse regions (Fig. 1). Statistical analysis indicated that DIXDC1 protein expression was significantly correlated with the histological intestinal-type (*P* < 0.001). DIXDC1 immunoreactivity was positively correlated with age (≥60 years, *P* < 0.001), the depth of invasion (*P* < 0.001) and the lymph node metastasis (*P* = 0.006). However, there was no significant correlation between DIXDC1 expression and other clinicopathological features such as gender (*P* = 0.086), size (≥5 cm,* P* = 0.141), tumor differentiation (*P* = 0.266) and clinical stage (*P* = 0.056) (Table 1).

**Table 1:** Correlation between DIXDC1 expression and clinicopathological parameters in 259 cases of gastric carcinoma

Clinicopathological feature	DIXDC1 + (n = 123)	DIXDC1 − (n = 136)	*P* value
Gender			0.086
Male	99	97	
Female	24	39	
Age			<0.001
≥60 years	77	52	
<60 years	46	84	
Size			0.141
≥5 cm	39	32	
<5 cm	84	104	
Differentiation			0.266
High grade	47	43	
Low grade	76	93	
Histological subtype			<0.001
Intestinal	97	54	
Diffused	13	66	
Mixed	12	13	
Indeterminate	1	3	
Depth of invasion			<0.001
T1	17	50	
T2	21	18	
T3 + T4	85	68	
Lymph node metastases			0.006
Negative	39	66	
Positive	84	70	
Clinical stage			0.056
I/II	70	93	
III/IV	53	43	

### Association between DIXDC1 expression and clinicopathological variables in intestinal-type and diffuse-type gastric carcinoma

DIXDC1 positive staining was observed more frequently in the intestinal-type of gastric carcinoma than in the diffuse-type. In the intestinal-type, but not in the diffuse-type, DIXDC1 positive expression was correlated with age (≥60 years, *P* = 0.018), size (≥5 cm, *P* = 0.036), tumor differentiation (*P* = 0.003), the depth of invasion (*P* < 0.001), the lymph node metastasis (*P* < 0.001) and the clinical stage (*P* = 0.003) (Table 2).

**Table 2:** Correlation between DIXDC1 expression and clinicopathological parameters in two types of gastric carcinoma

Clinicopathological feature	Intestinal-type gastric carcinoma			Diffuse-type gastric carcinoma		
	DIXDC1 + (n = 97)	DIXDC1 − (n = 54)	*P* value	DIXDC1 + (n = 13)	DIXDC1 − (n = 66)	*P* value
Gender			0.786			0.886
Male	79	43		8	42	
Female	18	11		5	24	
Age			0.018			0.072
≥60 years	59	22		8	23	
<60 years	38	32		5	43	
Size			0.036			0.563
≥5 cm	27	7		5	20	
<5 cm	70	47		8	46	
Differentiation			0.003			–
High grade	40	36		0	0	
Low grade	57	18		13	66	
Depth of invasion			<0.001			0.756
T1	13	29		3	18	
T2	18	6		1	9	
T3 + T4	66	19		9	39	
Lymph node metastases			<0.001			0.506
Negative	30	37		6	24	
Positive	67	17		7	42	
Clinical stage			0.003			0.650
I/II	56	44		7	40	
III/IV	41	10		6	26	

### Association of DIXDC1 with disease-specific survival

**Fig. 2:**
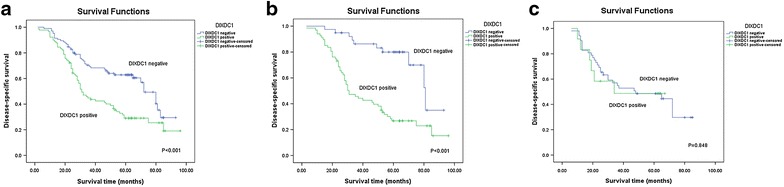
The relationships of DIXDC1 expression and the disease-specific survival rates. **a** A positive association of DIXDC1 expression and a decrease in DSS (n = 195); **b** in the intestinal-type of gastric carcinoma, the DIXDC1-positive group exhibited a lower DSS compared with the DIXDC1-negative group (n = 106); **c** in the diffuse-type of gastric carcinoma, no difference in DSS between the DIXDC1-positive and negative groups was observed (n = 64)

The relationships of DIXDC1 expression with the disease-specific survival rates were also analyzed. As shown in Fig. 2, patients with DIXDC1-positive expression had a significantly poorer prognosis than those without DIXDC1 expression (*P* < 0.001). In the intestinal-type of gastric carcinoma, the disease-specific survival rate of patients with DIXDC1-positive expression was also significantly lower than that of patients without DIXDC1 expression (*P* < 0.001, Fig. 2). However, no difference in disease-specific survival rate was observed between DIXDC1-positive and negative groups in the diffuse-type of gastric carcinoma (*P* = 0.848, Fig. 2).

 Univariate analysis of DSS in all cases revealed that, in addition to DIXDC1 expression (*P* < 0.001), age (*P* = 0.003), tumor size (*P* = 0.015), the depth of invasion (*P* < 0.001), lymph node metastasis (*P* < 0.001), and clinical stage (*P* < 0.001) were all associated with DSS (Table 3). The other clinicopathological features, such as gender (*P* = 0.561), histological subtype (*P* = 0.940) and tumor differentiation (*P* = 0.205), were not statistically prognostic factors. Multivariate analysis showed that DIXDC1 expression was an independent prognostic indicator in gastric carcinoma (HR = 0.674, *P* = 0.037) in addition to the depth of invasion (HR = 0.341, *P* = 0.011) and the clinical stage (HR = 0.272, *P* < 0.001) (Table 3). In the intestinal-type of gastric carcinoma, DIXDC1 expression was also a significant independent prognostic predictor (HR = 2.139, *P* = 0.036, Table 4). In addition, the depth of invasion also independently affected the disease-specific survival (HR = 2.153, *P* = 0.001, Table 4).

**Table 3:** Univariate and multivariate analysis in 259 cases of gastric carcinoma

Clinicopathological feature	Univariate	Multivariate		
	*P* value	HR	95 % CI	*P* value
Gender	0.561	–	–	–
Age	0.003	0.885	0.584–1.342	0.565
Size	0.015	0.941	0.618-1.433	0.778
Differentiation	0.205	–	–	–
Histological subtype	0.940	–	–	–
Depth of invasion	<0.001	0.341	0.149–0.781	0.011
Lymph node metastases	<0.001	1.053	0.566–1.959	0.871
Clinical stage	<0.001	0.272	0.151–0.491	<0.001
DIXDC1	<0.001	0.647	0.430–0.975	0.037

**Table 4:** Univariate and multivariate analysis in 151 cases of intestinal-type of gastric carcinoma

Clinicopathological feature	Univariate	Multivariate		
	*P* value	HR	95 % CI	*P* value
Gender	0.420	–	–	–
Age	0.004	1.588	0.904–2.787	0.108
Size	0.029	1.163	0.631–2.143	0.325
Differentiation	0.131	–	–	–
Depth of invasion	<0.001	2.153	1.377–3.368	0.001
Lymph node metastases	<0.001	1.372	0.731–2.577	0.325
Clinical stage	0.271	–	–	–
DIXDC1	<0.001	2.139	1.052–4.349	0.036

### Localization of β-catenin staining in gastric carcinoma

While our previous study showed that DIXDC1 enhances β-catenin nuclear localization in vitro [12], this has not been verified in gastric carcinoma tissues. Thus, additional cases were utilized to show the relationship between DIXDC1 and β-catenin by immunohistochemistry.

**Fig. 3:**
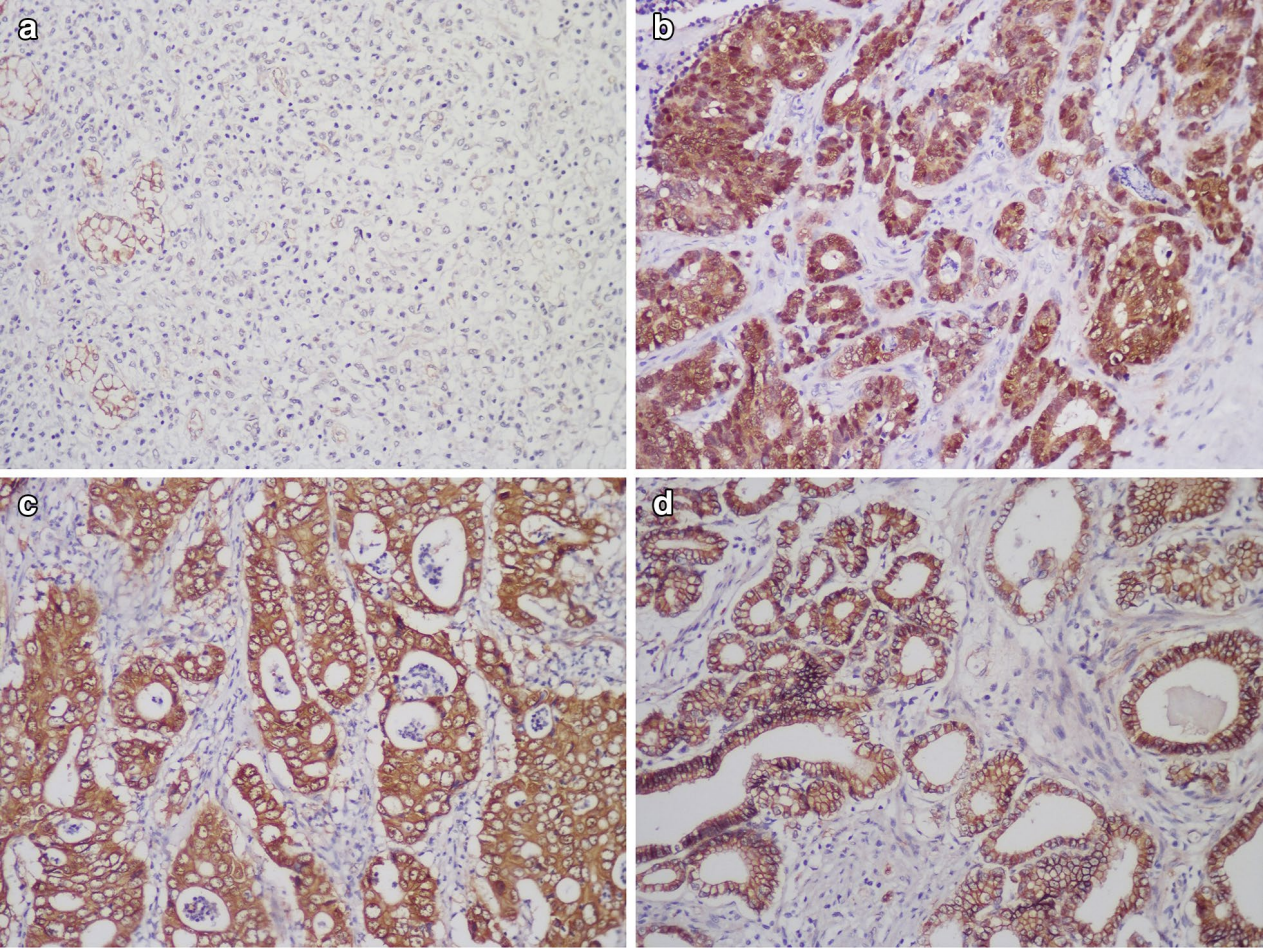
Localization of β-catenin staining in gastric carcinoma. In the diffuse-type group, tumor cells without expression of β-catenin, while the normal gastric epithelia exhibit continuous cell membrane staining (**a**). In the intestinal-type group, nuclear localization of β-catenin (**b**), cytoplasmic β-catenin staining (**c**), continuous membrane staining of β-catenin (**d**). (×200)

The immunohistochemical staining patterns of β-catenin were divided into 4 types: negative, membranous, cytoplasmic and nuclear staining patterns. Positivity with discontinuous or continuous distribution along the membrane or a very weak dotted expression were all included in the membranous pattern. We detected β-catenin expression using immunohistochemical staining in the 237 cases of gastric carcinoma mentioned above. In the normal gastric mucosa, β-catenin staining localized continuously to the cell membrane, while cytoplasmic and nuclear staining was generally absent (Fig. 3). As shown in Table 5, positive nuclear staining of β-catenin was observed in 75 (75/237, 31.6 %) cases of the total of 237 cases. Fifty-three cases exhibiting nuclear accumulation of β-catenin were intestinal-type gastric carcinoma (53/139, 38.1 %), while nuclear β-catenin localization was observed in 13 cases of diffuse-type (13/70, 18.6 %), 8 cases of mixed-type (8/24, 33.3 %), and 1 cases of indeterminate-type (1/4, 25.0 %). Cytoplasmic β-catenin staining was detected in 76 cases of gastric carcinoma (76/237, 32.1 %). Forty (40/139, 28.8 %) cases of intestinal-type and 29 (29/70, 41.4 %) cases of diffuse-type showed cytoplasmic expression of β-catenin. Membranous β-catenin staining was noted in 57 (57/237, 24.1 %) cases, while lost expression of β-catenin was observed in 29 (29/237, 12.2 %) cases (Fig. 3). The localization of β-catenin was not correlated with all histopathological subtypes of gastric carcinoma (*P* = 0.127) but was correlated with intestinal and diffuse-type (*P* = 0.025). Nuclear β-catenin expression was observed more frequently in the intestinal-type than in the diffuse-type.

**Table 5:** Localization of β-catenin staining in different histological subtypes of gastric carcinoma

β-catenin subtype	Nucleus ( %)	Cytoplasm	Membrane	Lost expression
Intestinal	53 (38.1)	40 (28.8)	31 (22.3)	15 (10.8)
Diffused	13 (18.6)	29 (41.4)	16 (22.9)	12 (17.1)
Mixed	8 (33.3)	5 (20.8)	9 (37.5)	2 (8.3)
Indeterminate	1 (25.0)	2 (50.0)	1 (25.0)	0 (0)

### The relationship between DIXDC1 expression and β-catenin localization in gastric carcinoma

**Fig. 4:**
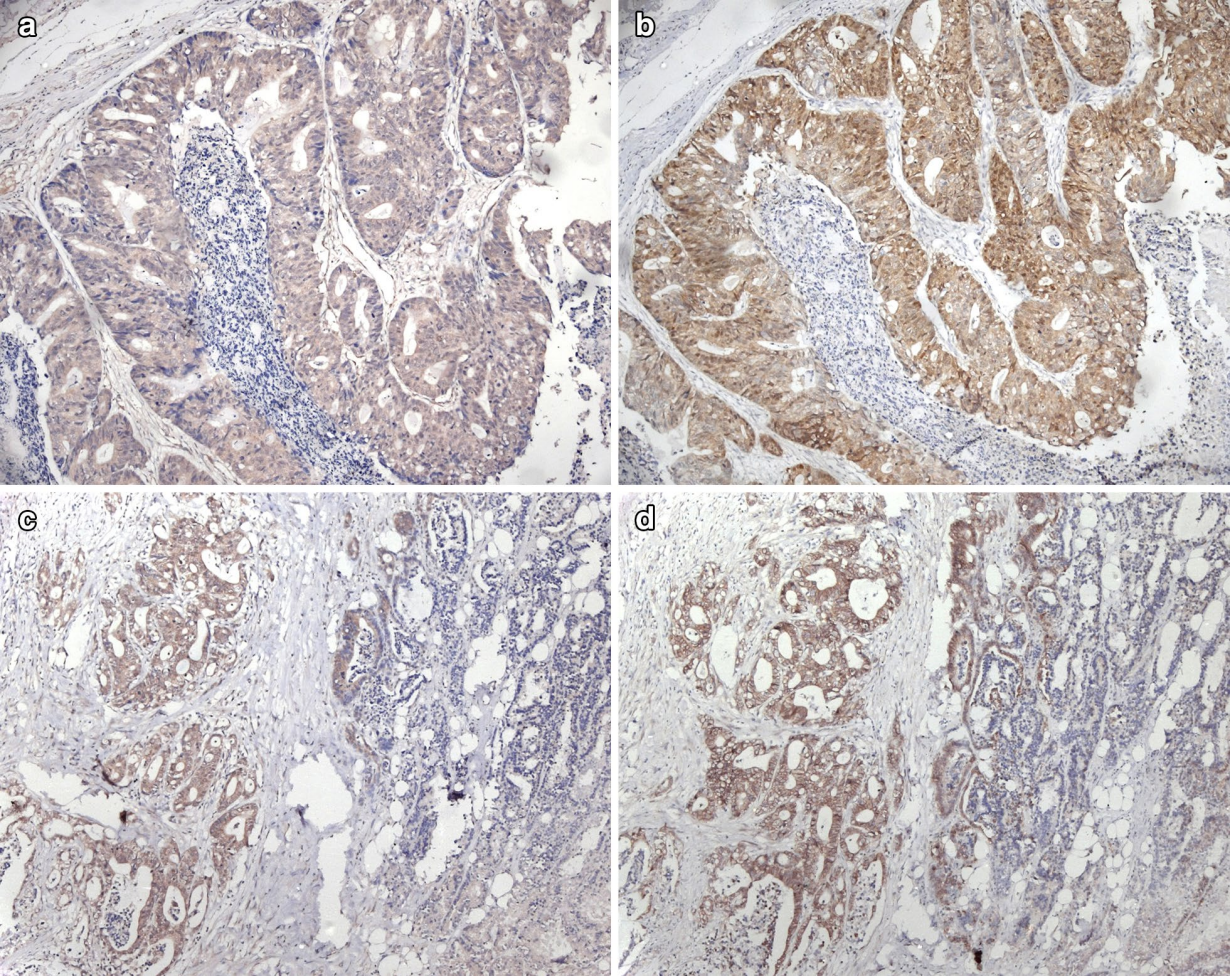
Co-localization of DIXDC1 and β-catenin expression in gastric carcinoma. Immunohistochemistry results showing strong DIXDC1 (**a**) and β-catenin staining (**b**) in the tumor area of the same case. (×200) In the case with heterogeneous DIXDC1 expression, strong β-catenin staining (**d**) was observed in the DIXDC1-positive staining area (**c**), while β-catenin was not observed in the area absent of DIXDC1 expression. (×100)

We then determined the correlation of DIXDC1 immunoreactivity with β-catenin expression pattern in gastric carcinoma. In DIXDC1-positive cases, nuclear β-catenin staining was observed in 43 cases (43/115, 37.4 %) and cytoplasmic β-catenin staining was observed in 33 cases (33/115, 28.7 %). In those DIXDC1-negative staining cases, nuclear β-catenin staining was observed in 32 cases (32/122, 26.2 %) and cytoplasmic β-catenin staining was observed in 43 cases (43/122, 35.2 %). However, no statistically significant association was observed between the DIXDC1 expression and the β-catenin localization (*P* = 0.243) in the cases overall. In intestinal-type gastric carcinoma, the patterns of β-catenin staining were significantly different between the cases with and without DIXDC1 expression (*P* = 0.002, Table 6). Nuclear and cytoplasmic expression of β-catenin were detected more frequently in the DIXDC1-positive expression group than in the DIXDC1-negative group. In addition, the immunohistochemistry results revealed that DIXDC1 and β-catenin co-localized well in some tissue specimens (Fig. 4). Even in cases with heterogeneous DIXDC1 expression, strong β-catenin expression was detected in the DIXDC1-positive staining area, while it was absent in the DIXDC1-negative staining area (Fig. 4).

**Table 6:** Relationship between DIXDC1 expression and localization of β-catenin staining in gastric carcinoma

β-catenin	Overall cases of gastric carcinoma			Intestinal-type of gastric carcinoma		
	DIXDC1 + (n = 115)	DIXDC1 − (n = 122)	*P* value	DIXDC1 + (n = 90)	DIXDC1 − (n = 49)	*P* value
Nucleus	43	32	0.243	37	16	0.002
Cytoplasm	33	43		37	10	
Membrane	24	33		13	21	
Lost expression	15	14		3	2

## Discussion

One of the most commonly used classifications of gastric carcinoma is the Lauren classification. According to the criteria of Lauren [18], gastric carcinomas are divided into two major categories: (a) intestinal-type or well-differentiated carcinoma growing primarily in glandular formation and (b) diffuse-type or poorly differentiated carcinoma primarily not forming glands and showing scattered cell growth with loose cell-to-cell adhesion. Tumors that contain approximately equal quantities of intestinal and diffuse components are called mixed-type. Carcinomas too undifferentiated to fit into each category are put into the indeterminate category (atypical type). The molecular alterations leading to these histological patterns are significantly different. The intestinal-type of gastric carcinoma is known to be very similar to that of colon carcinogenesis [1–4]. Mutations of the Wnt pathway genes APC and β-catenin can only be detected in a small portion of gastric carcinomas [6]. However, the mutations were observed more frequently in intestinal-type gastric cancer than in diffuse-type. Therefore, the canonical Wnt pathway was thought to participate in intestinal-type gastric carcinoma development and progression. However, E-cadherin mutation is a frequent genetic alteration in diffuse-type gastric cancer [19, 20].

Human DIXDC1 was identified as an Axin2 C-terminus interaction protein [21, 22]. The DIX domain is important for mediating multiple signal transductions [9, 23], and, in the Wnt signaling pathway, DIXDC1 is involved in both homomeric and heteromeric complexes between Axins and Dvl that form the multiprotein complexes of APC, GSK-3, and β-catenin, which regulate T-cell-factor (TCF) signaling [8, 9]. There have been a few studies on the biological role of human DIXDC1 so far. Previously, DIXDC1 was reported to be an Actin-binding protein and presumed to be connected with cell movement [21]. The overexpression of DIXDC1 can increase the invasion and migration ability of NSCLC [11]. Our previous study showed that overexpression of DIXDC1 can be observed in colorectal carcinoma and gastric carcinoma, and can increase the proliferation of colon cancer cells [10] and the invasion and migration ability of gastric cancer cells [12].

In this study, we expanded the number of gastric carcinoma samples to examine the correlation between DIXDC1 expression level and clinicopathological parameters, including patients’ prognoses. We found that positive expression of DIXDC1 can be detected in 47.5 % of gastric carcinoma. We also identified significant associations between DIXDC1 expression and the depth of tumor invasion (T), lymph node metastasis (N) and disease-specific survival, which suggested that DIXDC1 plays an important role in gastric carcinoma. Additionally, we found that the expression of DIXDC1 was significantly correlated with histological subtype of gastric carcinoma, and it was more commonly detected in the intestinal-type (64.2 %) than in the diffuse-type (16.5 %). In the intestinal-type group, DIXDC1 expression was associated with age (≥60 years), tumor size (≥5 cm), differentiation, the depth of invasion, lymph node metastasis and clinical stage. However, there was no correlation between DIXDC1 expression and the clinicopathological parameters mentioned above in the diffuse-type group. Similar to our previously published data [12], we found that patients with DIXDC1-positive expression had significantly poorer prognoses than those without DIXDC1 expression in allcases. However, the above result was observed in the intestinal-type group but not in the diffuse-type. As a result of expanding the sample size, we found that DIXDC1 may play an important role in carcinogenesis and progression of the intestinal-type of gastric carcinoma.

Cytoplasmic and/or nuclear localization of β-catenin can be used to estimate the activation of the Wnt pathway. The expression patterns of β-catenin in gastric carcinoma tissue were detected in this study, and the results showed that β-catenin nuclear localization was detected more frequently in the intestinal-type than in the diffuse-type. In contrast to colon cancer, mutation of β-catenin in gastric carcinoma is not as well-defined. The incidence of β-catenin mutations was significantly different in different studies [3, 4, 24–29]. The mechanisms regulating the Wnt signaling pathway in gastric carcinoma remained to be elucidated. Our previous in vitro experiments showed that DIXDC1 interacted with β-catenin, inhibited the phosphorylation of β-catenin and increased the translocation of β-catenin to the nucleus in a gastric carcinoma cell line, indicating that DIXDC1 could positively regulate the Wnt signaling pathway [12]. In this study, we analyzed the co-localization of DIXDC1 and β-catenin in the tumor specimens to verify our in vitro results. Cytoplasmic/nuclear localization of β-catenin was observed more frequently in the cases with positive DIXDC1 expression than those without DIXDC1 expression in intestinal-type gastric carcinoma. In addition, there was a very robust co-localization of DIXDC1 and β-catenin in the tissue specimens. Heterogeneous expression of DIXDC1 was observed in some areas of the gastric cancer specimen, and β-catenin expression was also detected in these areas, which further validated our in vitro experiments.

## Conclusions

The present study reveals, to our knowledge for the first time, that the expression of DIXDC1 is correlated with the histological subtype of gastric carcinoma, and DIXDC1 may participate in the activation of Wnt in the carcinogenesis and progression of the intestinal-type of gastric tumor. DIXDC1 is associated with biologically more aggressive phenotypes of gastric carcinoma, and DIXDC1-positive expression is an independent biomarker for poor prognosis, especially in intestinal-type gastric carcinoma.

## Methods

### Tumor specimens and clinical data collection

The study was approved by The Clinical Research Ethics Committee of Fudan University Shanghai Cancer Center. Written informed consents were obtained from the patients. We collected 259 cases of primary gastric carcinoma from the files of the Department of Pathology, Fudan University Shanghai Cancer Center. Patients who suffered from familial cancer syndromes or were previously treated with chemotherapy were not included. All specimens were routinely processed and stained with hematoxylin and eosin (H&E) for histological examination. All cases were reviewed by two pathologists and the histological diagnoses were confirmed without discrepancy. All cases were classified according to the Lauren classification as intestinal-type (n = 151), diffuse-type (n = 79), mixed-type (n = 25) or indeterminate-type (n = 4). Clinicopathological parameters, such as gender, age at the time of diagnosis, tumor size, the depth of invasion, differentiation, lymph node metastasis and clinical stage, were obtained from the medical records of the patients. A total of 195 patients were followed up after the surgery until April 30th, 2015 and were included in the survival analysis. The disease-specific survival (DSS) was defined as the length of time between the surgery and death, specifically from the cancer.

### Immunohistochemical staining

Representative paraffin blocks containing tumor and normal mucosa from each case were selected for immunohistochemical analysis of DIXDC1 and β-catenin expression. Four μm-thick sections of the paraffin-embedded tissues were cut and deparaffinized according to standard histological techniques. Endogenous peroxidase activity was blocked by soaking the slides in 0.3 % H_2_O_2_ in methanol for 30 min at 37 °C. Antigen retrieval was performed by high pressure repair in 0.1 M citrate solution (pH 6.0) for 10 min followed by incubation with anti-DIXDC1 polyclonal antibody (Cat. No. sc-292126, Santa Cruz Biotechnology, USA) diluted at 1:100 or anti-β-catenin monoclonal antibody (Cat. No. 610154, BD Biosciences, USA) diluted at 1:200. After an overnight incubation in primary antibodies at 4 °C, the slides were washed with PBS and then incubated for 60 min at room temperature with the biotinylated link antibody (Cat. No. K0609, Universal LSABTM2Kit/HRP, Rabbit/Mouse, Dako A/S, Denmark) diluted at 1:200. After being washed with PBS, the sections were stained with 0.05 % diaminobenzidine containing 0.01 % hydrogen peroxidase. Finally, the sections were counterstained with hematoxylin, dehydrated and mounted. Omission of the primary antibody and substitution by non-specific immunoglobins were used as negative controls.

### Immunohistochemical evaluation

The sections were evaluated by two pathologists who were blinded to the clinical outcomes. The staining of DIXDC1 was coded as positive or negative (scored as 0). The positive cases were divided into weak (scored as 1), moderate (scored as 2) and strong (scored as 3) based on the staining intensity. The score was calculated by adding the multiplication of the different staining intensities in the four gradations with each percentage of positive cells. The mean score for the duplicate scores from each pathologist was calculated for a final score from 0 to 300 points. A tumor sample having a score ≥50 points was considered positive [30]. The staining patterns of β-catenin were divided into four types: (1) lost expression; (2) membranous pattern, positivity with discontinuous or continuous distribution along the membrane or a very weak dotted expression [31]; (3) cytoplasmic pattern; and (4) nuclear pattern, when β-catenin staining was observed in the nuclei of more than 10 % of the tumor cells, expression was considered positive for nuclear staining, even if it was previously reported to be associated with cytoplasmic and/or membranous staining [32].

### Statistical analysis

The χ2 test and the Fisher’s test were used to test correlations between DIXDC1 expression, β-catenin localization, and clinicopathological parameters, such as histological type, gender, age, tumor size, the depth of invasion, differentiation and lymph node metastasis. The χ2 test was also used for analyzing correlations between β-catenin expression pattern and histopathological subtype of gastric carcinoma. Univariate analyses were performed using the Kaplan–Meier method, and the survival difference between groups was assessed by the log-rank test. Variables with a value of *P* < 0.05 were selected for multivariate analysis. Multivariate analysis was performed using the Cox proportional hazards regression model. Differences were considered to be significant at *P* < 0.05. SPSS (Chicago, IL) version 18.0 was used to analyze all of the data.

## Abbreviations

DIXDC1: Dishevelled-Axin domain containing 1
GC: gastric carcinoma
Ccd1: Coiled-coil-Dishevelled-Axin1
DIX: Dishevelled-Axin
APC: adenomatous polyposis coli
GSK-3β: glycogen synthase kinase 3 beta
TCF: T cell-factor
NSCLC: non-small cell lung cancer
H&E: hematoxylin and eosin
DSS: disease-specific survival
